# A Pro-resolving Role for Galectin-1 in Acute Inflammation

**DOI:** 10.3389/fphar.2020.00274

**Published:** 2020-03-20

**Authors:** Hannah L. Law, Rachael D. Wright, Asif J. Iqbal, Lucy V. Norling, Dianne Cooper

**Affiliations:** ^1^The William Harvey Research Institute, Barts and The London School of Medicine and Dentistry, Queen Mary University of London, London, United Kingdom; ^2^Institute of Translational Medicine, University of Liverpool, Liverpool, United Kingdom; ^3^Institute of Cardiovascular Sciences, College of Medical and Dental Sciences, University of Birmingham, Birmingham, United Kingdom; ^4^Centre for Inflammation and Therapeutic Innovation, Queen Mary University of London, London, United Kingdom

**Keywords:** galectin-1, leukocytes, inflammation, resolution, apoptosis

## Abstract

Galectin-1 (Gal-1) exerts immune-regulatory and anti-inflammatory actions in animal models of acute and chronic inflammation. Its release into the extracellular milieu often correlates with the peak of inflammation suggesting that it may serve a pro-resolving function. Gal-1 is reported to inhibit neutrophil recruitment and induce surface exposure of phosphatidylserine (PS), an “eat me” signal on the surface of neutrophils, yet its role in resolution remains to be fully elucidated. We hypothesized that the anti-inflammatory and pro-resolving properties of Gal-1 are mediated through its ability to inhibit neutrophil recruitment and potentiate neutrophil clearance. To investigate this, a murine model of self-resolving inflammation was utilized to uncover the role of both the endogenous and exogenous protein using Gal-1 null mice and recombinant protein, respectively. We found that peritoneal macrophages express increased Gal-1 during the resolution phase and enhanced neutrophil recruitment occurs in the early phases of zymosan peritonitis in Gal-1 null mice compared to their wild-type (WT) counterparts. Administration of recombinant Gal-1 following the peak of inflammation led to reduced neutrophil numbers at 24 and 48 h, shortening the resolution interval from 39 to 14 h. Gal-1 treatment also enhanced neutrophil apoptosis, indicating a pro-resolving action. Together these results indicate an important role for Gal-1 in the timely resolution of acute inflammation.

## Introduction

Neutrophil trafficking to the site of inflammation is essential for the clearance of infection and repair of injured tissue. However, excessive inflammation is deleterious to the host and can result in damage to healthy tissue and the development of chronic inflammatory pathologies such as rheumatoid arthritis and atherosclerosis (Nathan and Ding, 2010; Perretti et al., 2017; Kasikara et al., 2018). It is now widely accepted that the resolution of inflammation is an active process, driven by the generation of pro-resolving mediators such as resolvins and Annexin A1 by infiltrated neutrophils (Sugimoto et al., 2019). These mediators promote resolution through the inhibition of neutrophil trafficking, induction of neutrophil apoptosis, and promotion of neutrophil clearance through efferocytosis, processes that are key to the restoration of tissue homeostasis (Norling and Perretti, 2013).

Galectin-1 (Gal-1) is the prototype member of the galectin family, which shares a specificity for β-galactoside containing proteins and lipids. Several galectins have been ascribed immunomodulatory functions, with Gal-1 generally regarded as anti-inflammatory due to its inhibitory effects on neutrophil and T cell trafficking and induction of T cell apoptosis (He and Baum, 2006; Toscano et al., 2007; Cooper et al., 2008; Norling et al., 2008; Earl et al., 2010). Evidence is emerging that Gal-1 may also have pro-resolving actions: expression often peaks at the height of the inflammatory response (Ilarregui et al., 2009; Iqbal et al., 2011; Starossom et al., 2012) and Gal-1 was identified in resolving exudates of mice in a model of peritonitis and was downregulated by the resolution-toxic anesthetic lidocaine (Chiang et al., 2008). Current findings suggest that Gal-1 links the innate and adaptive immune systems as differentiation of dendritic cells in a Gal-1 rich environment leads to enhanced regulatory functions and suppression of autoimmune disease progression (Ilarregui et al., 2009). However, more recent evidence has expanded the pro-resolving potential of Gal-1 to cells of the innate immune system. Gal-1 has been shown to induce 12/15-lipoxygenase expression in macrophages, switching them to a pro-resolving phenotype (Rostoker et al., 2013). A potential role for Gal-1 in neutrophil clearance has also been suggested due to its ability to induce phosphatidylserine (PS) exposure on the surface of neutrophils *in vitro* (Stowell et al., 2007); however, evidence of this *in vivo* is currently lacking.

In this study, we challenge the hypothesis that alongside its recognized anti-inflammatory actions, Gal-1 also possesses pro-resolving properties through its actions on neutrophil trafficking and lifespan. Using a well-characterized model of zymosan-induced peritonitis, we demonstrate that in the absence of Gal-1 there is increased neutrophil recruitment to the peritoneal cavity, while administration of recombinant Gal-1 after the peak of inflammation induces neutrophil apoptosis and clearance.

## Materials and Methods

### Galectin-1

Recombinant Human Gal-1 (hrGal-1) was provided by GalPharma (Takamatsu, Kagawa, Japan). The recombinant protein is a cysteine-less mutant with all cysteine residues substituted with serine residues, which is resistant to oxidation while retaining all known activities of native Gal-1 (Nishi et al., 2008).

### Ethics and Regulations

Experiments performed *in vivo* adhered to Home Office regulations (Scientific Procedures Act, 1986) and were additionally approved under the guidelines set down by the Ethical Committee for the Use of Animals, Barts and The London School of Medicine. Additionally, protocols under the supervision of the above were accomplished baring careful consideration to the principles set out by the National Centre for Replacement Refinement and Reduction of Animals in Research (NC3Rs).

### Mice

Male wild-type (WT) C57/BL6 mice were purchased from Charles River (Kent, United Kingdom). Original breeding pairs of Gal-1 knockout (KO) mice (*Lgals1* null) animals were generously provided by the Consortium for Functional Glycomics^1^ and were bred and housed at Charles River (Kent, United Kingdom). Where required WT and KO mice were both age and sex-matched. Mice were housed within individually ventilated cages (IVCs), a maximum of six mice per cage in a facility with a 12 h light–dark cycle and *ad libitum* feeding of a standard laboratory chow diet and water.

### Zymosan-Induced Peritonitis Model

Zymosan-induced peritonitis was performed with Gal-1 KO mice and WT counterparts or with WT mice administered hrGal-1 (10 μg). Mice aged 6–8 weeks (*n* = 3–8) were administered zymosan [1 mg in 500 μl Dulbecco’s phosphate buffered saline (DPBS^+/+^)] by intraperitoneal (i.p) injection at the 0 h time point. Mice treated with hrGal-1 were given a dose of 10 μg or vehicle (200 μl DPBS^+/+^) only control i. p at 8 h post-zymosan (during the peak phase of response). Mice were sacrificed at the indicated time points. Peritoneal cavities were lavaged with ice cold DPBS^–/–^ containing 2 mM EDTA. Total cell numbers in the peritoneum were quantified by cell counts using Turk’s Solution. Cell free lavage fluid was retained for analysis of Gal-1 or inflammatory cytokine levels by ELISA (R&D systems, Abingdon, United Kingdom, and Labospace Milan, respectively). For *ex vivo* apoptosis experiments, mice were treated with hrGal-1 at 2 h and lavaged at 6 h, cells from 1 ml peritoneal exudate were then resuspended in RPMI + 0.5% bovine serum albumin (BSA) and incubated for 20h at 37°C in 5% CO_2_ prior to analysis.

### Flow Cytometry

Differential cell counts in the peritoneal cavity as well as intracellular Gal-1 levels were assessed by flow cytometry. Leukocyte infiltrate was quantified using CD45^+^ cells as the parent population and the subsequent percentage of positive cells by biomarker as follows. Immune cells were labeled with the following panel of fluorescently conjugated antibodies: CD45 PerCP (clone 30-F11, Biolegend), F4/80 BV650 (clone BM8, Biolegend) or F4/80 APC (clone BM8, eBioscience), Ly6G PE (clone 1A8, BD Pharmingen), 7/4 FITC (clone 7/4, Abcam), CD11b BV785 (clone M1/70, Biolegend) and SiglecF (clone ES22-10D8, Mitenyl Biotech). In some instances, Ly6C PerCP-Cy5.5 (clone HK1.4, eBioscience) was used. Following antibody incubations, cells were washed twice with FACS buffer before fixation in 1% paraformaldehyde (PFA) solution.

For Gal-1 expression cells were stained as above for cell specific markers (Ly6C, Ly6G, and F4/80) and then fixed and permeabilized with BD fixation and permeabilization buffer before addition of anti-Gal-1 antibody (polyclonal, R&D systems) followed by AF488 anti-goat IgG (polyclonal, Invitrogen) secondary antibody to assess Gal-1 levels. In all cases, antibodies or relevant isotype controls were incubated for a minimum of 30 min at 4°C prior to analysis on a BD LSR Fortessa (BDbiosciences) and analyzed post acquisition using FlowJo (v10) software.

For *in vivo* neutrophil apoptosis, the peritoneal exudate was analyzed immediately post collection. Pelleted cells from 200 μl peritoneal exudate were stained with Ly6G (as above). Cells were washed twice, followed by the addition of AnnexinV FITC (BD Pharmingen) and Zombie NIR (Biolegend) in AnnexinV binding buffer (BD Pharmingen). Cells were resuspended, covered, and incubated [15 min, at room temperature (RT)]. AnnexinV binding buffer was added to each sample and cells were analyzed immediately by flow cytometry. Quadrant gating was applied to determine viable (AnxV^–^NIR^–^), early apoptotic (AnxV^+^NIR^–^), late apoptotic (AnxV^+^NIR^+^), and necrotic (AnxV^–^NIR^+^) neutrophil populations.

### Statistical Analysis

Data are expressed as mean ± SEM. Comparisons were analyzed for statistical significance. GraphPad Prism (v7) software was used for all analyses and a *P* ≤ 0.05 was considered as statistically significant. Levels of Gal-1 profiled across time (0, 4, 24, and 48 h) were analyzed using a one-way ANOVA with Tukey’s multiple comparisons test. Leukocyte numbers profiled across time (2, 6, 24, and 48 h) were analyzed using a two-way repeated measures ANOVA with Sidak’s multiple comparisons test. Leukocyte numbers at a single time point (24 h) and percentage of neutrophils in each quadrant for apoptosis were analyzed using an unpaired *t*-test.

## Results

### Macrophages Are a Source of Gal-1 in the Peritoneal Cavity

**FIGURE 1:**
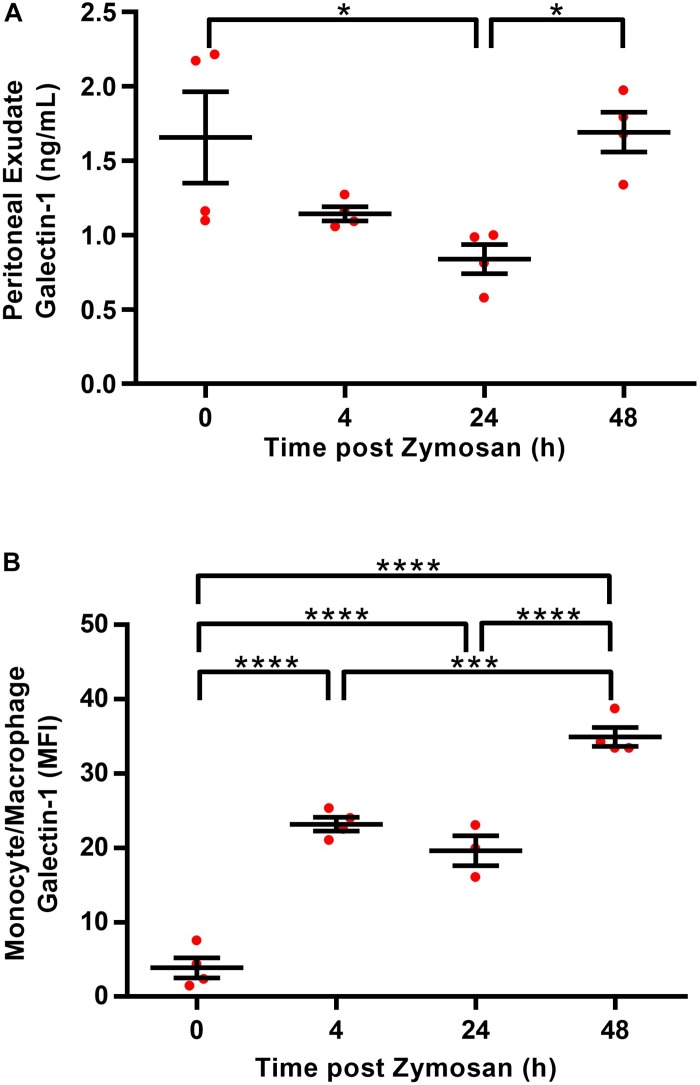
Macrophages are a source of Gal-1 in the peritoneal cavity. Peritoneal lavages were performed on C57/Bl6 naive mice (0 h) or 4, 24, and 48 h following administration of zymosan (1mg in 500 μl DPBS^+/+^ i.p.) and cell free supernatants were assayed for Gal-1 concentration by ELISA **(A)**. Total Gal-1 levels were assessed in permeabilized monocytes/macrophages (identified using Ly6C^+^, F4/80^+^) by flow cytometry **(B)**. Statistical analysis was performed using a one-way ANOVA with Tukey’s multiple comparisons test, results are displayed as mean ± SEM, and in all cases, significant results are considered as *P < 0.05, with ***P < 0.0005 and ****P < 0.0001. *n* = 3–4 mice per group.

Given that reports in the literature indicate that Gal-1 levels typically peak at the height of inflammation, we initially sought to characterize endogenous expression of Gal-1 over the course of a self-limiting model of zymosan peritonitis in WT mice. Levels were assessed in both the exudate and the immune cells (monocytes/macrophages and neutrophils) within the cavity. Surprisingly, Gal-1 was readily detected in the peritoneal cavity of naive mice (Figure 1A); however, levels were comparatively low in the resident macrophage population at this time point (Figure 1B) suggesting a non-immune cell source of the protein in naive mice. Levels of Gal-1 within the exudate decreased following the onset of inflammation with around a 50% reduction at 24 h post-zymosan. A second peak was observed at 48 h post-zymosan (Figure 1A), which correlated with increased levels in the monocytes/macrophages (Ly6C^+^, F4/80^+^) (Figure 1B). Gal-1 expression was negligible in the neutrophil (Ly6G^+^) population (data not shown).

### Leukocyte Recruitment Is Enhanced in Gal-1 KO Mice

**FIGURE 2:**
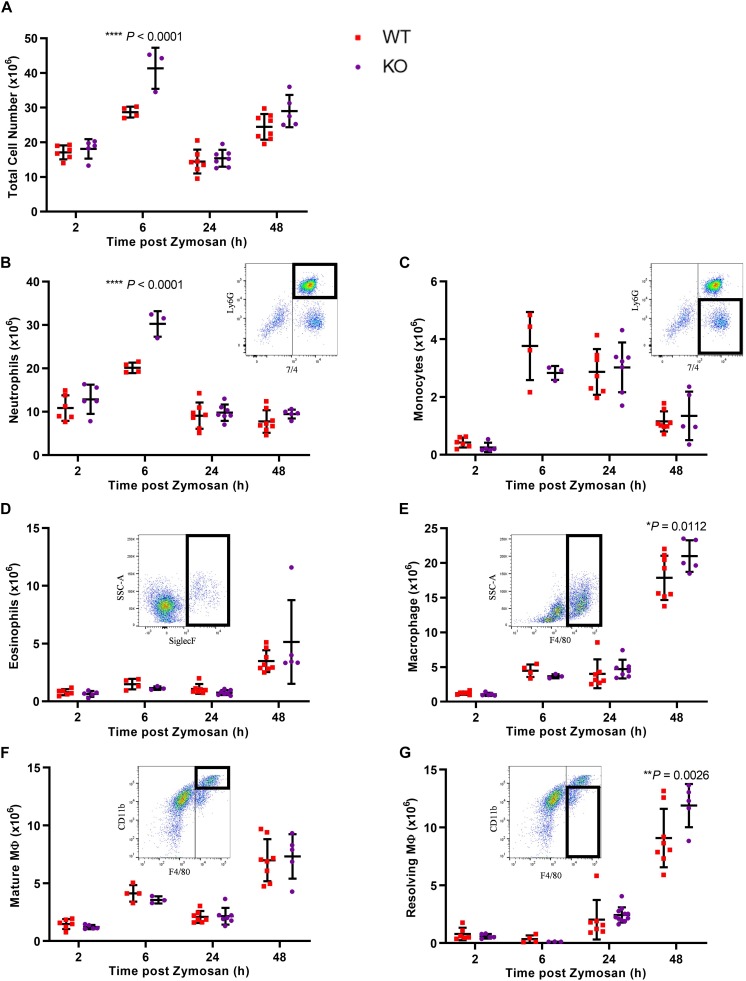
Leukocyte recruitment is enhanced in Gal-1 KO mice. Mice received zymosan (1 mg in 500 μl DPBS^+/+^ i.p.) and peritoneal lavage was performed at 2, 6, 24, and 48 h. Total cell counts were performed **(A)** and the number of neutrophils (7/4^+^Ly6G^+^) **(B)**, inflammatory monocytes (7/4^+^Ly6G^–^) **(C)**, eosinophils (Siglec F^+^) **(D)**, macrophages (F4/80^+^) **(E)**, mature macrophages (F4/80^+^CD11b^high^) **(F)**, and resolving macrophages (F4/80^+^CD11b^low^) **(G)** were identified by flow cytometry. Representative flow cytometry plots are shown for each subset. Statistical analysis was performed using a two-way ANOVA with Sidak’s multiple comparisons test, results are displayed as mean ± SEM, and in all cases, significant results are considered as *P* < 0.05. *n* = 3–8 mice per group.

Given the increased levels of Gal-1 in the peritoneal cavity during the resolution phase and its expression in the macrophage population within the cavity, we next sought to determine whether the absence of Gal-1 would modulate the inflammatory profile induced by zymosan by utilizing Gal-1 KO mice. In WT mice, there was a sharp and significant increase in leukocyte influx into the peritoneal cavity from 2 to 6 h as expected, followed by a decline at 24 h. A biphasic response was observed with a second increase in total leukocyte number at 48 h, as shown in Figure 2A. Leukocyte recruitment in Gal-1 KO mice mirrored that observed in WT mice in terms of the temporal nature of the response; however, significantly more leukocytes migrated into the peritoneal cavities of Gal-1 KO mice compared to WT at the peak of the inflammatory response (6 h time point).

Further analysis of leukocyte subtypes identified neutrophils (7/4^+^Ly6G^+^) as the predominant cell type from 2 h onward, with a decline in number observed at 24 h. While this trend was observed in both genotypes, significantly more neutrophils trafficked to the peritoneal cavity of Gal-1 KO mice, compared to WT at the 6 h time point as shown in Figure 2B.

Monocyte (7/4^+^Ly6G^–^) numbers displayed a bell-shaped trend (Figure 2C) with low numbers at 2 h, a respective rise at 6 and 24 h followed by a decline at 48 h in WT mice. No differences were observed between the genotypes. Eosinophil (Siglec F^+^) numbers rose sharply at 48h post-zymosan, indicative of their pro-resolving phenotype (Yamada et al., 2011), with no significant differences between genotypes (Figure 2D). In WT mice, macrophage (F4/80^+^) numbers were lowest at 2 h, increased from 2 to 6 h and remained unchanged at 24 h before rising sharply at 48 h (Figure 2E). At early time points, there was no significant difference between macrophage numbers in the cavities of Gal-1 KO mice compared to WT; however, significantly more macrophages were present in Gal-1 KO mice at 48 h. Macrophage phenotype was also assessed and while there were no significant differences between genotypes in the number of mature macrophages (F4/80^+^CD11b^high^), significantly more resolving macrophages (F4/80^+^CD11b^low^) were present within the cavities of Gal-1 KO mice at 48 h compared to their WT counterparts (Figures 2F,G).

Along with increased neutrophil recruitment in Gal-1 KO mice, we also detected significant increases in the pro-inflammatory cytokine IL-6 as well as the pro-angiogenic growth factor VEGF in KO mice, with a trend toward increased levels of the chemokines CCL2, CCL3, and CXCL1 and the pro-inflammatory cytokine TNF-α (Supplementary Figures S1A–L).

### hrGal-1 Administration Following the Peak of Inflammation Promotes Neutrophil Clearance

**FIGURE 3:**
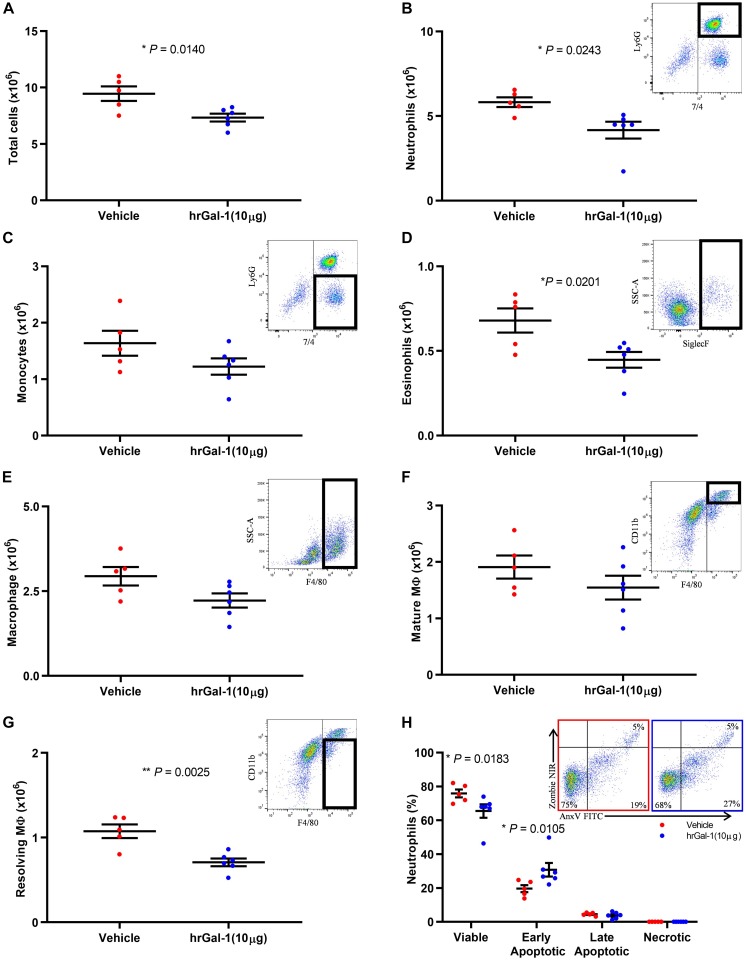
hrGal-1 administration following the peak of inflammation promotes neutrophil clearance. C57/Bl6 mice received zymosan (1 mg in 500 μl DPBS^+/+^ i.p.) at 0 h. At 8 h post-zymosan mice were administered hrGal-1 (10 μg) or vehicle (200 μl DPBS^+/+^) i.p. and peritoneal lavage performed at 24 h. Total cell counts were performed **(A)**. Flow cytometry was used to analyze the cell population for leukocyte subsets including neutrophils (7/4^+^Ly6G^+^) **(B)**, inflammatory monocytes (7/4^+^Ly6G^–^) **(C)**, eosinophils (Siglec F^+^) **(D)**, macrophages (F4/80^+^) **(E)**, mature macrophages (F4/80^+^CD11b^high^) **(F)**, and resolving macrophages (F4/80^+^CD11b^low^) **(G)**. Representative flow cytometry plots are inset for each subset. The neutrophil (Ly6G^+^) population was further assessed by AnnexinV (FITC) and Zombie (NIR). Quadrant gating was applied to determine viable (AnxV^–^NIR^–^), early apoptotic (AnxV^+^NIR^–^), late apoptotic (AnxV^+^NIR^+^), and necrotic (AnxV^–^NIR^+^) neutrophil populations. Results for the percentages of the neutrophil population within each of the quadrants are shown **(H)** with representative flow cytometry plots inset for vehicle (left) and hrGal-1 (right) treated mice. Statistical analysis was performed using an unpaired t test (with each quadrant for apoptosis), results are displayed as the mean ± SEM, and in all cases, significant results are considered as *P* < 0.05. *n* = 5–6 mice per group.

Given the enhanced neutrophil recruitment observed in Gal-1 KO mice, we next addressed whether Gal-1 is able to drive resolution when administered following the peak of an inflammatory response. To this end, hrGal-1 was administered i.p. at 8 h post-zymosan, a time point where neutrophil trafficking will have peaked in this model. Administration of hrGal-1 at this time point resulted in significantly fewer leukocytes being recovered 16 h later (24 h time point) compared to those recovered from vehicle treated mice (Figure 3A). As shown in Figures 3B–G, further analysis of the individual leukocyte subtypes indicated that there was a trend toward an all-round decrease in the numbers of all leukocyte subsets analyzed in the cavities of mice treated with hrGal-1 (a trend that was also observed at 48 h, see Supplementary Table S1). Significant reductions in the numbers of neutrophils (7/4^+^Ly6G^+^; Figure 3B), eosinophils (SiglecF^+^; Figure 3D), and resolving macrophages (F4/80^+^CD11b^low^; Figure 3G) were observed.

Neutrophils that were collected from the peritoneal cavity at 24 h post-zymosan injection were further assessed for apoptosis. The majority of neutrophils were viable (70.69 ± 5.21%), presumably as a result of the rapid clearance of apoptotic cells. Importantly, significantly fewer viable cells were detected within the peritoneal cavities of mice treated with hrGal-1 compared to vehicle (Figure 3H), and a significant increase in the percentage of early apoptotic neutrophils (AnxV^+^/PI^–^) was observed. The percentage of late apoptotic cells was minimal in both genotypes again presumably due to their clearance, with negligible numbers of necrotic neutrophils detected.

To further address the effect of Gal-1 on the apoptotic process, hrGal-1 was administered during the initial recruitment phase (2 h) and peritoneal lavages performed at 6 h post-zymosan. Cells were then incubated *in vitro* overnight to assess apoptosis. Here we found that *ex vivo* late apoptosis of neutrophils was significantly increased following exposure to Gal-1 (as illustrated in Supplementary Figure S2).

### hrGal-1 Administration Following the Peak of Inflammation Shortened the Resolution Interval

**FIGURE 4:**
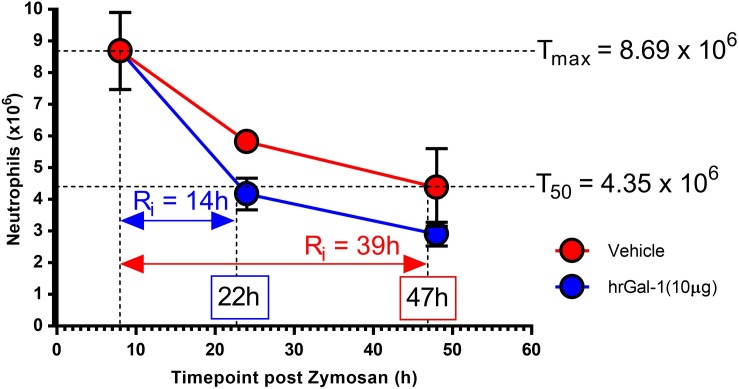
hrGal-1 administration following the peak of inflammation shortened the resolution interval. Peritonitis was initiated in C57/Bl6 mice using zymosan (1 mg in 500 μl DPBS^+/+^ given i.p.) and mice were treated with hrGal-1 (10 μg) or vehicle (200 μl DPBS^+/+^) by i. p injection 8 h post-zymosan. Peritoneal lavages were performed at 8, 24, and 48 h. Total cell counts were performed and flow cytometry was used to quantify neutrophil (7/4^+^Ly6G^+^) numbers. Resolution indices were calculated by monitoring the maximum peritoneal neutrophil number (T_max_), when neutrophil numbers declined by 50% (T_50_) and the resolution interval (R_i_), the time from T_max_ to T_50_. *n* = 5–6 mice per group.

In order to quantitate the pro-resolving actions of Gal-1, resolution indices were calculated. These are widely utilized to identify mediators that stimulate, as well as those that disrupt or delay resolution (Bannenberg et al., 2005; Schwab et al., 2007; Navarro-Xavier et al., 2010). Parameters calculated included the amplitude and duration of the inflammatory response by monitoring the maximum neutrophil infiltrate (T_max_), when a 50% loss in neutrophil numbers occurred (T_50_) and the resolution interval (R_i_; time from T_max_ to T_50_). Treatment with hrGal-1 shortened the resolution interval from 39 h observed in vehicle treated mice to 14 h, indicating that Gal-1 accelerated neutrophil clearance from the peritoneal cavity (Figure 4).

## Discussion

Leukocyte recruitment is an integral component of the inflammatory response, whereby a mass migration of cells through the vasculature toward the site of inflammation occurs. In this context, it is well established that neutrophils are the primary leukocyte subset mobilized. While neutrophil recruitment is critical for a successful inflammatory response, the temporal and spatial confinement of the neutrophilic infiltrate is crucial for resolution. Apoptosis is a critical step in paving the way for successful resolution of acute inflammation, where apoptotic neutrophils play an essential role in reprogramming macrophages toward a pro-resolving state (Fredman et al., 2012). Our findings suggest that Gal-1 should be included in the expanding list of pro-resolving molecules.

Gal-1 was readily detectable in peritoneal lavage fluid, with the highest levels detected at 0 and 48 h, suggesting levels are highest in the absence of inflammation and during resolution. Macrophages have previously been identified as a source of Gal-1, with levels highest in M2 macrophages in the peritoneal cavity (Rabinovich et al., 1996; Fredman et al., 2012), which correlates with the significant expression observed in macrophages at 48 h in this current study.

Our data indicate the effects of Gal-1 are multi-faceted, with neutrophil trafficking and clearance impacted by either the lack of, or addition of excess (exogenous) Gal-1. Neutrophil trafficking is significantly enhanced in the absence of Gal-1, a result that corroborates with the increased leukocyte emigration observed in the microcirculation of Gal-1 KO mice (Cooper et al., 2008) and corresponds with reports in the literature of an inhibitory role for Gal-1 on neutrophil recruitment (Iqbal et al., 2011). In line with a heightened inflammatory response in the absence of Gal-1, IL-6 and VEGF levels were also significantly increased in Gal-1 KO mice. IL-6 is a pleiotropic cytokine involved in the acute phase response and its inhibition has been shown to reduce the inflammatory response in a model of zymosan peritonitis (Cuzzocrea et al., 1999) while VEGF has been shown to induce rapid recruitment of neutrophils *in vivo* (Massena et al., 2015).

The majority of studies indicating an effect of Gal-1 on neutrophil trafficking have focused on the actions of the exogenous protein (La et al., 2003). Indeed, in a previous study from our laboratory, administration of hrGal-1 reduced neutrophil infiltration in a model of carageenan-induced paw edema; however, the edema profile was unchanged in the first 24 h post-carageenan in Gal-1 KO mice (Iqbal et al., 2011). The enhanced neutrophil infiltration observed in Gal-1 KO in the current study may indicate context specific actions for the endogenous protein with increased neutrophil trafficking also observed in Gal-1 KO mice in the colon in response to infection with Citrobacter *rodentium* (Curciarello et al., 2014).

Inflammation induced by zymosan is reliant on the activation of peritoneal macrophages via TLR2; evidence exists in the literature that exogenous Gal-1 reduces pro-inflammatory cytokine production from murine macrophages (Fredman et al., 2012; Abebayehu et al., 2017) and skews toward an M2 (Barrionuevo et al., 2007) and pro-resolving phenotype (Fredman et al., 2012). Additionally, macrophages from Gal-1 KO mice have been shown to have enhanced expression of MHCII (Barrionuevo et al., 2007). We have seen increased levels of pro-inflammatory mediators in the early recruitment phase of the inflammatory response in Gal-1 KO mice despite reduced macrophage numbers in the peritoneal cavity, which may be suggestive of a heightened pro-inflammatory response in macrophages in the absence of Gal-1. To the best of our knowledge, a full characterization of Gal-1 KO macrophage phenotype has not been performed and the exact role of endogenous Gal-1 in macrophages still requires full characterization.

While significantly more neutrophils were found within the peritoneal cavity of Gal-1 KO mice at 6 h, this difference was lost at 24 h suggesting that clearance might be enhanced in these mice. Assessment of apoptosis and efferocytosis at this time point showed no differences in the percentage of cells undergoing either process (data not shown). It is feasible that the apoptotic/efferocytic process is faster or earlier in Gal-1 KO mice, or alternatively these results may simply be a reflection of the efficiency of the resolution process and the ability of the system to regain homeostasis. Exogenous Gal-1 has been demonstrated to enhance macrophage phagocytosis of sheep red blood cells *in vitro*; however, interestingly Gal-1 KO macrophages also phagocytosed more effectively than WT cells when cultured *ex vivo* (Barrionuevo et al., 2007). More recently, Gal-1 was shown to induce efferocytic satiation in a model of zymosan-induced peritonitis which is linked to early departure of these macrophages from the resolving cavity. Surprisingly, although Gal-1 is thought to enhance macrophage infiltration into the resolving peritoneum (Gil et al., 2006; Malik et al., 2009), we did not see a reduction in macrophage numbers in the peritoneal cavity of Gal-1 KO animals, in fact the adverse was evidenced. This may be in response to the increased neutrophil infiltrate observed in these mice. These findings propose a divergence in the roles of endogenous and exogenous Gal-1 and thus further work is required to fully understand its role in efferocytosis.

There are several studies describing the ability of hrGal-1 to inhibit neutrophil trafficking both *in vitro* and *in vivo*; however, its effects on neutrophil clearance have not, to the best of our knowledge been assessed *in vivo*. Importantly, in this experimental setup, we determined neutrophil numbers in response to exogenous Gal-1 administration after the peak of inflammation (at a time point that should not affect neutrophil recruitment) in order to assess neutrophil clearance. *In vitro* studies from Stowell et al. (2009, 2007) describe the ability of Gal-1 to induce exposure of the “eat me” signal PS on the surface of neutrophils. Interestingly, PS exposure normally occurs as part of the early stages of apoptosis; however, Gal-1 was not found to induce apoptosis in their studies, rather it was proposed that Gal-1-induced PS exposure is a mechanism to drive clearance of neutrophils in the absence of apoptosis. Here, we detected increased numbers of early apoptotic neutrophils within the peritoneal cavities of mice treated with hrGal-1. While it was not possible to determine whether these cells progressed through the apoptotic process presumably due to the rapid clearance of PS expressing cells *in vivo*, we did uncover a pro-apoptotic role for Gal-1 when assessing the stages of apoptosis *ex vivo*. Further studies are required to elucidate the mechanisms behind this pro-apoptotic effect of Gal-1.

In support of the rapid engulfment of PS exposing neutrophils in this model the lower numbers of resolving macrophages detected 16 h post Gal-1 administration is suggestive of their quick retreat to the lymphatics. Findings by Rostoker et al. demonstrated that hrGal-1 treatment converts macrophages from a CD11b^high^ to a CD11b^low^ phenotype during resolution. The shift toward a resolving phenotype could be a direct response to the increased numbers of early apoptotic neutrophils that we report within our study. As hrGal-1 increases PS exposure on neutrophils, an action that has been linked to enhanced clearance (Stowell et al., 2009), this “eat me” signal might stimulate the macrophage phenotypic switch to better enable clearance of these cells. We propose that hrGal-1 treated mice undergo macrophage phenotype switching sooner as a result of the increased apoptotic neutrophils present in the cavity and the requirement of their clearance. We hypothesize that the consequence of the more rapid engulfment of apoptotic neutrophils is that resolving macrophages in hrGal-1 treated mice reach efferocytic satiation sooner and thus depart the cavity more swiftly.

There is evidence in the literature that Gal-1 can induce apoptosis of human synovial fluid neutrophils from rheumatoid arthritis patients (Cedeno-Laurent et al., 2010), which may indicate a divergence between the effects of Gal-1 on neutrophils in the periphery versus those that have trafficked to the inflammatory site. Neutrophils that have trafficked to the inflammatory site are known to express a different repertoire of receptors (Hartl et al., 2008) and it is likely that upon transmigration, Gal-1 is able to bind to a newly expressed receptor on the cell surface. This also correlates with studies indicating increased galectin binding to activated neutrophils and importantly for this study on neutrophils that have trafficked to the inflammatory site (Almkvist et al., 2002). Furthermore, the glycosylation status of neutrophils is modulated upon transmigration, particularly with respect to sialic acid residues (Cross et al., 2003). Sialic acid capping of glycoproteins negatively impacts Gal-1 binding (Toscano et al., 2007), thus a reduction in terminal sialic acids, through the action of sialidases, may also permit the increased binding of Gal-1 to neutrophils post-transmigration. The effects of galectins are indeed highly complex and vary depending on local concentration, intracellular or extracellular localization, or differentiation status of the target cell, which may account for effects on neutrophils in the periphery as well as in tissue.

The reduction in the resolution interval in response to Gal-1 in this model provides further indication that the protein augments the transition from inflammation to resolution. Gal-1 administration at the 8 h time point reduced the resolution interval from 39 to 14 h, suggesting that Gal-1 has pro-resolving properties that may be of therapeutic utility. While native Gal-1 is susceptible to oxidation, which can negatively impact its activity, the form utilized in this study has been modified to prevent oxidation and extend the half-life of the protein (Nishi et al., 2008) suggesting that modified forms of the protein may be therapeutically advantageous. However, due to the complexity of galectin biology, a further understanding of their mechanism of action is required before galectin-based therapeutics can be utilized clinically.

Collectively, our findings confirm an anti-trafficking role for endogenous Gal-1 and indicate a pro-apoptotic function for the exogenous soluble protein. The clearance of apoptotic cells is a fundamental process following cell damage, aging, and trafficking of leukocytes to an inflammatory site and hence is critical for the resolution of inflammation and preservation of self-tolerance. Our results enhance our understanding of the role of Gal-1 in resolution and sheds light on the promising nature of this protein for the development of a new class of therapeutics that activate resolution.

## Data Availability Statement

The datasets generated for this study are available on request to the corresponding author.

## Ethics Statement

The animal study was reviewed and approved by the Queen Mary University of London Local Ethical Review Committee and in accordance with the United Kingdom Home Office regulations (Guidance on the Operation of Animals, Scientific Procedures Act, 1986).

## Author Contributions

DC and LN planned the project. DC and AI provided the knockout mice. HL, RW, and AI performed and analyzed the experiments. HL, RW, LN, and DC contributed to the design of the experiments. HL, LN, and DC wrote and edited the manuscript.

## Conflict of Interest

The authors declare that the research was conducted in the absence of any commercial or financial relationships that could be construed as a potential conflict of interest.

## Footnotes

1 http://www.functionalglycomics.org

## Supplementary Material

FIGURE S1 Inflammatory mediators are increased in the absence of Gal-1. Mice received zymosan (1 mg in 500 μl DPBS^+/+^ i.p.) and peritoneal lavage was performed at 2 h. Exudate was assessed for total cell counts **(A)** and the number of neutrophils (7/4^+^Ly6G^+^) **(B)**, eosinophils (Siglec F^+^) **(C)**, inflammatory monocytes (7/4^+^Ly6G^–^) **(D)**, mature macrophages (F4/80^+^CD11b^high^) **(E)**, and resolving macrophages (F4/80^+^CD11b^low^) **(F)** were identified by flow cytometry. The cell free supernatant was collected from 1ml of peritoneal exudate and concentrations of analytes measured by ELISA. Results for the concentrations of cell recruitment chemokines CCL2 (MCP-1) **(G)**, CCL3 (MIP-1-α) **(H)**, and CXCL-1 (KC) **(I)** are shown. Levels of inflammatory cytokines TNF-α **(J)** and IL-6 **(K)** are shown for both genotypes, as well as the growth factor VEGF **(L)**. Statistical analysis was performed using an unpaired *t*-test and significant results considered as *P* < 0.05, *n* = 7–8 mice per group.

FIGURE S2 *Ex vivo* neutrophil apoptosis is increased with hrGal-1. C57/Bl6 mice received zymosan (1 mg in 500 μl DPBS^+/+^ i.p.) at 0 h. At 2 h post-zymosan mice were administered hrGal-1 (10 μg) or vehicle (200 μl DPBS^–/–^) and peritoneal lavage performed at 6 h to collect leukocytes from the cavity. Peritoneal exudate (1 ml) cells were resuspended in RPMI–1640 Medium + 0.5% BSA and incubated (20 h). Flow cytometry was used to select the neutrophil (Ly6G^+^) population which was further assessed by AnnexinV (FITC) and Zombie (NIR). Quadrant gating was applied to determine viable (AnxV^–^NIR^–^), early apoptotic (AnxV^+^NIR^–^), late apoptotic (AnxV^+^NIR^+^), and necrotic (AnxV^–^NIR^+^) neutrophil populations in vehicle **(A)** and hrGal-1 **(B)** treated mice. Results for the percentages of the neutrophil population within each of the quadrants are shown **(C)**. Statistical analysis was performed using an unpaired *t-*test (with each quadrant for apoptosis), results are displayed as the mean ± SEM, and in all cases, significant results are considered as *P* < 0.05. *n* = 6–7 mice per group.

TABLE S1 Leukocyte numbers in 48 h peritoneal exudate with hrGal-1 administered following the peak of inflammation. Peritonitis was initiated in C57/Bl6 mice using zymosan (1 mg, i.p.) and mice were treated with hrGal-1 (10 μg) or vehicle (200 μl DPBS^–/–^) 8 h post-zymosan and peritoneal lavage was performed at 48 h. Total cell counts were performed and the number of neutrophils (7/4^+^Ly6G^+^), inflammatory monocytes (7/4^+^Ly6G^–^), eosinophils (SiglecF^+^), macrophages (F4/80^+^), mature macrophages (F4/80^+^CD11b^high^), and resolving macrophages (F4/80^+^CD11b^low^) were identified by flow cytometry. Statistical analysis was performed using an unpaired *t*-test. Results displayed as the mean ± SEM; in all cases, significant results were considered as *P* < 0.05. *n* = 5 mice per group.
