# Supplementary figures and images for: A Pro-resolving Role for Galectin-1 in Acute Inflammation
Source: Front Pharmacol. 2020 Mar 20;11:274. doi: 10.3389/fphar.2020.00274 (PMC7098973; doi:10.3389/fphar.2020.00274)

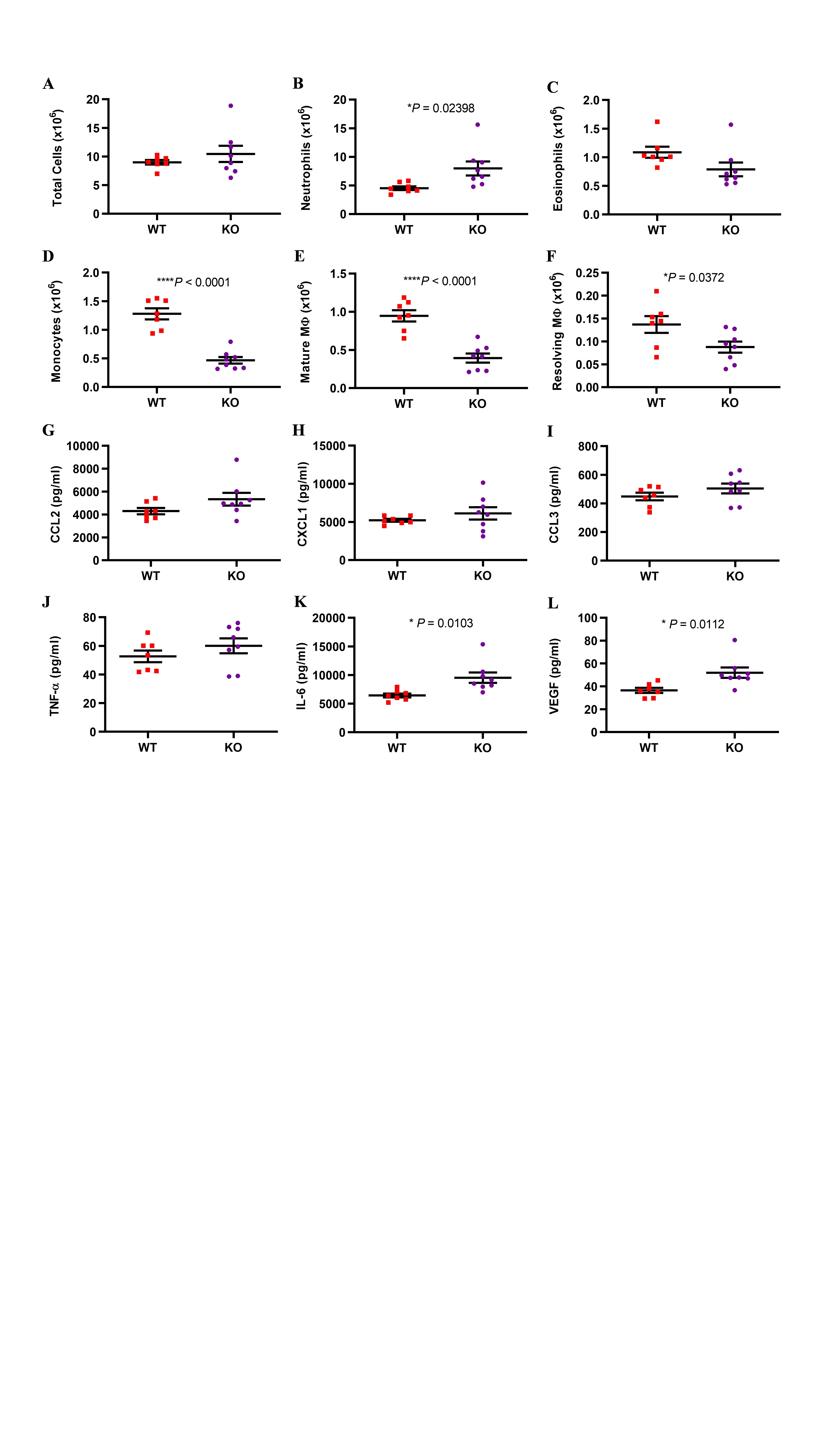

Supplement: FIGURE S1 — Inflammatory mediators are increased in the absence of Gal-1. Mice received zymosan (1 mg in 500 μl DPBS+/+ i.p.) and peritoneal lavage was performed at 2 h. Exudate was assessed for total cell counts (A) and the number of neutrophils (7/4+Ly6G+) (B), eosinophils (Siglec F+) (C), inflammatory monocytes (7/4+Ly6G–) (D), mature macrophages (F4/80+CD11bhigh) (E), and resolving macrophages (F4/80+CD11blow) (F) were identified by flow cytometry. The cell free supernatant was collected from 1ml of peritoneal exudate and concentrations of analytes measured by ELISA. Results for the concentrations of cell recruitment chemokines CCL2 (MCP-1) (G), CCL3 (MIP-1-α) (H), and CXCL-1 (KC) (I) are shown. Levels of inflammatory cytokines TNF-α (J) and IL-6 (K) are shown for both genotypes, as well as the growth factor VEGF (L). Statistical analysis was performed using an unpaired t-test and significant results considered as P < 0.05, n = 7–8 mice per group. [file Image_1.JPEG]

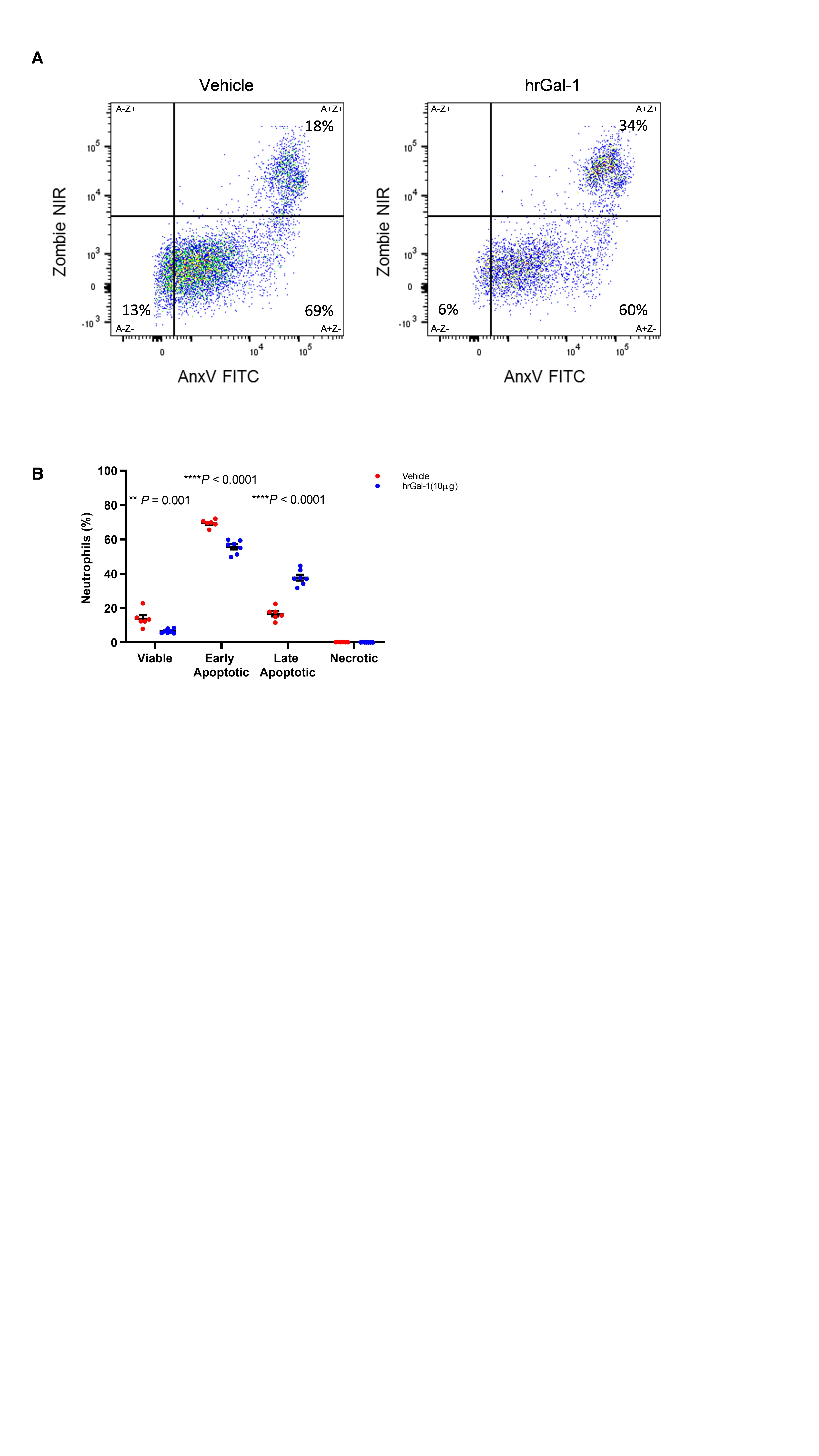

Supplement: FIGURE S2 — Ex vivo neutrophil apoptosis is increased with hrGal-1. C57/Bl6 mice received zymosan (1 mg in 500 μl DPBS+/+ i.p.) at 0 h. At 2 h post-zymosan mice were administered hrGal-1 (10 μg) or vehicle (200 μl DPBS–/–) and peritoneal lavage performed at 6 h to collect leukocytes from the cavity. Peritoneal exudate (1 ml) cells were resuspended in RPMI–1640 Medium + 0.5% BSA and incubated (20 h). Flow cytometry was used to select the neutrophil (Ly6G+) population which was further assessed by AnnexinV (FITC) and Zombie (NIR). Quadrant gating was applied to determine viable (AnxV–NIR–), early apoptotic (AnxV+NIR–), late apoptotic (AnxV+NIR+), and necrotic (AnxV–NIR+) neutrophil populations in vehicle (A) and hrGal-1 (B) treated mice. Results for the percentages of the neutrophil population within each of the quadrants are shown (C). Statistical analysis was performed using an unpaired t-test (with each quadrant for apoptosis), results are displayed as the mean ± SEM, and in all cases, significant results are considered as P < 0.05. n = 6–7 mice per group. [file Image_2.JPEG]
