# Supplementary material for: A Pro-resolving Role for Galectin-1 in Acute Inflammation
Source: Front Pharmacol. 2020 Mar 20;11:274. doi: 10.3389/fphar.2020.00274 (PMC7098973; doi:10.3389/fphar.2020.00274)
Supplement: TABLE S1 — Leukocyte numbers in 48 h peritoneal exudate with hrGal-1 administered following the peak of inflammation. Peritonitis was initiated in C57/Bl6 mice using zymosan (1 mg, i.p.) and mice were treated with hrGal-1 (10 μg) or vehicle (200 μl DPBS–/–) 8 h post-zymosan and peritoneal lavage was performed at 48 h. Total cell counts were performed and the number of neutrophils (7/4+Ly6G+), inflammatory monocytes (7/4+Ly6G–), eosinophils (SiglecF+), macrophages (F4/80+), mature macrophages (F4/80+CD11bhigh), and resolving macrophages (F4/80+CD11blow) were identified by flow cytometry. Statistical analysis was performed using an unpaired t-test. Results displayed as the mean ± SEM; in all cases, significant results were considered as P < 0.05. n = 5 mice per group. [file Table_1.DOCX]

A pro-resolving role for Galectin-1 in acute inflammation

| Leukocytes (x 10^6^) | **Vehicle** | **hrGal-1 (10µg)** |
| --- | --- | --- |
| **Total Cells** | 12.9 ± 2.50 | 7.05 ± 1.28 |
| **Neutrophils** | 4.38 ± 1.23 | 2.91 ± 0.38 |
| **Monocytes** | 2.17 ± 0.57 | 0.98 ± 0.18 |
| **Macrophages** | 5.33 ± 1.57 | 2.32 ± 0.46 |
| **Mature Macrophages** | 3.90 ± 1.26 | 1.43 ± 0.26 |
| **Resolving Macrophages** | 1.45 ± 0.32 | 1.05 ± 0.23 |
| **Eosinophils** | 2.12 ± 0.55 | 1.44 ± 0.14 |

**Supplementary Table 1. Leukocyte numbers in 48h peritoneal exudate with hrGal-1 administered following the peak of inflammation.** Peritonitis was initiated in C57/Bl6 mice using zymosan (1mg, i.p.) and mice were treated with hrGal-1 (10µg) or vehicle (200µl DPBS-/-) 8h post-zymosan and peritoneal lavage was performed at 48h. Total cell counts were performed and the number of neutrophils (7/4+Ly6G+), inflammatory monocytes (7/4+Ly6G-), eosinophils (SiglecF+), macrophages (F4/80+), mature macrophages (F4/80+CD11bhigh) and resolving macrophages (F4/80+CD11blow) were identified by flow cytometry. Statistical analysis was performed using an unpaired t test. Results displayed as the mean ± SEM, in all cases significant results were considered as P < 0.05. n=5 mice per group.
